# The Conservation of Motherhood

**Published:** 1920-10-30

**Authors:** 


### The Conservation of Motherhood.

### HOW TO PREVENT WASTE OF ENERGY.

There seems now to be scarcely any sphere of life in which some aspect of motherhood is not an important consideration. Even the British Commercial Gas Association, at their conference a few days ago at Sheffield, could not prevent it from cropping up with great insistence when they discussed the gas industry as a factor in national economy. Mrs. M. A. Cloudesley-Brereton made the question the dominant element of her striking address on the conservation of energy. In the factories, she pointed out, we see every labour-saving device used—incidentally that is putting the case rather high, but the contrast between the factory and the average home is certainly impressive and depressing. Household work is human work; women do not ask that it should be less human, but that it should be more humane. The modern world expects more and more from womanhood, but a mother can only give of her best with the sympathetic co-operation of her husband, and also with all the help that modern progress can devise.

At present the wastage of human energy is enormous—such, for instance, as that involved by the carrying of water from the top to the bottom of houses, the universal method of the slums. All new houses, Mrs. Brereton urged, should at least be fitted with gas-fires in bedrooms and parlours. The use of gas appliances would go far to solve the servant problem. Twenty years ago in America gas heating and cooking apparatus was made part of the equipment of a workman's house. and American women who come to this country as units are perfectly aghast at the condition of things in the workmen's homes here. In America women have discovered how to help women, and Mrs. Brereton's hope is that British women will insist that in the new houses now contemplated and planned a gas-cooker is put at the start as part of the original building and not as an extra.

Mrs. Brereton also touched upon a relevant matter which has been discussed more than once in these columns, and that is how the children of the present day are being educated in domestic things.

"We ought to be very clear on the point," she said, "that girls don't get the kind of teaching in their own homes nowadays that they used to get in domestic matters, because we have rather got it into our heads that it is the teachers' business in the schools, and we are leaving that duty to them. I think it is up to us to inquire what kind of teaching is given in the school and to see that the girls stay long enough to get domestic training. I was talking the other day to a headmistress to whom a mother came and said, 'I'll thank you not to teach my girl to cook and darn, and all that kind of thing, because she's going to be a lady.' That is a horrible attitude. It is the kind of thing that makes people look down upon servants." And the kind of thing, we will add, that makes servants look down upon an honourable calling, and adds tenfold to the perplexities and hardships in the administration of the modern home. Mrs. Brereton paid high testimony to what she termed the vicarious and altruistic motherhood of our medical and teaching professions. One of the most valuable bodies of women in the kingdom to-day are the teachers of domestic economy in all their various capacities.

With regard to woman's life in industry, she pointed out that there is still a heavy economic loss associated with labour turnover—the amount of change of staff which is necessary to keep up staff strength. A turnover of 100 per cent. is quite common—that is to say, that to keep a staff of 100 up to strength a hundred new workers must be engaged every year. The annual waste is enormous—one eminent authority has at a conservative estimate computed it to be £16,000,000 for factories. What is more difficult to estimate is the cost in human energy. Some proportion of the turnover is no doubt due, as was stated by the lecturer, to a faulty selection of workers in the first place, but conditions also are a considerable element. The human body works best when at a temperature most suitable to its immediate and varying conditions, and means must be found to vary that temperature with the varying needs. Medical evidence suggests that the best heating is afforded by modern gas appliances, because it allows of the best ventilation. But for a manual as well as for a mental worker bodily comfort cannot be divorced from comfort of the mind. Dr. Edgar Collis, one of the foremost authorities on industrial health, recently predicted that the nineteenth century would be famous for the study of the machine and the first half of the twentieth century for the study of mankind. A woman at any rate is now expected to be something far more than useful about the house—she is "the repository of our national trust, the safeguard of our national destiny."

